# Improving Learning Outcomes: The iPad and Preschool Children with Disabilities

**DOI:** 10.3389/fpsyg.2017.00660

**Published:** 2017-05-05

**Authors:** Linda Chmiliar

**Affiliations:** Centre for Social Sciences, Athabasca University, AthabascaAB, Canada

**Keywords:** iPads, preschool, disabilities, tablets, touch screen

## Abstract

The digital age has reached early childhood, and the use of touch screens by young children is common place. Research on the use of touch screen tablets with young children is becoming more prevalent; however, less information is available on the use of touch screen tablets to support young children with disabilities. Touch screen tablets may offer possibilities to preschool children with disabilities to participate in learning in a digital way. The iPad provides easy interaction on the touch screen and access to a multitude of engaging early learning applications. This paper summarizes a pilot study with 8 young children with disabilities included in a preschool classroom, who were given iPads to use in class and at home for a period of 21 weeks. Systematic observations, classroom assessments, and teacher and parent interviews documented the improvements in learning outcomes for each child in many areas including, but not limited to: shape and color recognition, letter recognition, and tracing letters throughout six research cycles.

## Introduction

The digital age is upon us, and with ownership of mobile devices increasing in families, many young children now have access to the use of use touch screen tablets. There is considerable debate in the mainstream media as to whether or not young children benefit from the use of these technologies, or whether these devices are harmful. Research on the use of mobile devices with young children is growing, but information on how to use these devices with young children with disabilities is still relatively scarce. Touch screen tablets offer possibilities to preschool children with disabilities such as the ability to explore learning in a digital way. The iPad provides easy interaction on the touch screen and access to a multitude of intuitive, engaging learning applications. The cognitive ability required to use this technology is substantially lower than for traditional digital technologies and even young children with disabilities can learn how to use this tool quite quickly (Chmiliar, 2013). This paper summarizes a pilot study that examined the use of iPads by eight preschool children with a range of disabilities included in a preschool classroom. The children received iPads to use at school and a home for a period of 21 weeks. This qualitative research study documented the learning each child demonstrated at home and at school; parent and teacher perceptions of the use of the iPad by each child; the use of the iPad in the classroom; and the supports that the parents and educators needed to use the iPad effectively.

A number of studies in the literature have indicated that the use of computer technologies with young children can be beneficial and can provide children with an opportunity to learn and practice skills in an engaging and interactive environment. Roschelle et al. (2000) found that the use of computer-based technologies can be very simulating and motivating for young children. Hitchcock and Noonan (2000) found that computer assisted instruction of early academic skills was successful in improving skills. Johnson et al. (2010) studied 180 preschool and kindergarten children and reported positive changes in skills when using a computer-assisted instruction, particularly with linear sequenced materials. Li and Atkins (2004) reported that early computer exposure during preschool years was associated with the development of concepts and cognition. Children who use computers have been found to show greater gains in intelligence, structural knowledge, problem solving, and language skills compared with those who do not use technology in their learning (Clements and Samara, 2003; Swaminathan and Wright, 2003; Vernadakis et al., 2005).

Research on newer technologies such as iPods and iPad with preschool children has emerged. A number of these studies have looked at the use of these devices to promote literacy skills. Dobler (2012) in classroom of first graders observed that young children were able to successfully work together for literacy practice with limited teacher assistance. Beschorner and Hutchison (2013) used six iPads in two preschool classrooms of 4- and 5-year-old children over a 7-week period of time. Apps focused on classroom skills were loaded on the iPads biweekly. They found that the children could navigate and use the iPads independently. They also observed that the children developed emergent literacy skills using the device. Students could manipulate magnet letters to write their and their friends names and several students could identify letters and use the keyboard to write simple stories and books. In a case study of two preschool classrooms with 3–5years old, Flewitt et al. (2014) looked at the use of iPads for literacy activities. Their results demonstrated that literacy activities on the iPad stimulated children’s motivation and concentration. The preschool staff-recognized the potential for learning with the iPads and observed increased concentration in task completion, and enhanced communication and collaboration. Wong (2015), in a year-long qualitative study with 3–5 years old, found that young children can use iPads to communicate and learn. Children in the study were observed to gain literacy knowledge.

There have been several studies that explored the use of iPads for drawing and printing with preschool children. Couse and Chen (2010) explored the viability of tablet computers in early education, by investigating preschool children’s ease in acclimating to tablet technology and its effectiveness in engaging them to draw. A total of 41 3- to 6-year-old children were videotaped while they used the tablets. The study found significant differences in level of tablet use between sessions. The teachers reported high child interest in the task and the drawings produced by the children were typical to above expectation. Matthews and Seow (2007), in a small descriptive study, looked at the symbolic representation of 12 children ages 2–11 years using electronic paint on tablet computers. The researchers videotaped children drawing with both tablet computers and traditional media. Although they reported similarities in the children’s drawings using both types of media, they found that the tablet and stylus-interfaced technology was a superior tool for drawing. Patchan and Puranik (2016) looked at the use of iPads to teach preschool children how to write letters. They found that the haptic feedback provided by using a finger on the iPad to write letters helped young children learn how to write. They noted that using a finger was better than a stylus.

The use of iPads for play has also been explored with young children. Verenikina and Kervin (2011) looked at the potential for digitally mediated imaginative play with the iPad. They conducted case studies of three families with preschool children and found that the children were able to engage in imaginative play on the iPad. Murdock et al. (2013) examined the use of an app on the iPad to improve play. Three of the four children in the study demonstrated moderate and sustained improvements in play dialog that was independently generated.

Several studies have examined the used of the iPad into the everyday activities of the preschool classroom. Clark and Abbott (2016) looked at how the iPads impacted learning in literacy, numeracy and learning skills in a primary school. Improvements and greater readiness in the student’s ability to learn concepts in literacy and numeracy were observed by the teacher for all students including those with lower ability and special needs. They also found that motivation, concentration and confidence grew. Another classroom-based research study (Kucirkova et al., 2014), looked at the effect of a story making app on iPads in a preschool classroom. They found that the children’s engagement was higher with the story making app.

Although there is evidence in the literature regarding the use of iPads with young children, there is less information regarding the use of the iPad with young children with disabilities. Lee (2015) looked at the use of iPads in a case study of preschool children age three to five enrolled in two different preschool classrooms in a Head Start Program. A number of children had behavioral difficulties, some were English Language Learners, and several had hearing and speech impairments. The results indicated that the use of the iPad resulted in enhanced interactions between the children and the apps supported development. The children found the apps to be fun and higher levels of engagement and higher levels of motivation were reported. Another study also focused on children in Head Start programs. Brown and Harmon (2013), in a pilot study, looked at the efficacy of iPad applications in improving the literacy and overall academic skills in at-risk preschoolers. Their study included 24 children from five different Head Start classrooms. After a post-test on alphabet knowledge, matching, and number concepts, they reported that use of the iPad-supported learning in the areas of alphabet knowledge and number concepts. Zhen et al. (2015) looked at the effects of using an iPad application to teach four young children with disabilities to identify initial phonemes and found that performance was improved and the children enjoyed using the iPad. Chmiliar (2013, 2014), in a series of two pilot studies with preschool children with disabilities, found that young children between the ages of three to five were able to successfully learn to navigate the iPad. In each of the pilot studies, preschool children with a range of disabilities used iPads independently at home over an 8-week period of time. The children demonstrated improvements in many preschool skills at the conclusion of the study. For example: many of the children learned to print their name using a tracing app, several children learned to count to 100, most of the children improved their ability to complete puzzles, and one child started to talk saying words specifically related to an app about trains on the iPad. Chai et al. (2015) examined the use of an iPad application with children with developmental delays to teach early literacy skills. They found that all of the students were able to learn the target phonemes and were able to generalize the skills across materials.

Several studies were found that looked at the use of iPads with young children with autism. Vandermeer et al. (2012) examined the use of social stories on the iPad to increase on-task behavior and attention with one 5-year-old girl with autism. The child demonstrated an interest in using the iPad and an increase in attention at the end of the study. Kemp et al. (2016) found that two young children with autism spectrum disorders were better engaged in media with iPad apps than with picture books. Other studies focused primarily on the use of the iPad to promote language. Ganz et al. (2013) in a study of three children ages three to four with autism, looked at the use of a picture exchange communication system (PECS) on the iPad compared to a traditional PECS. The PECS on iPad was as effective as the traditional picture system, and two of the three children preferred to use the app system on the iPad instead of the traditional PECS. Lorah et al. (2014) looked at sentence frame discrimination using the iPad with young children with developmental disabilities and autism spectrum disorder. They had success training students to use the iPads as a speech generating device for labeling. King et al. (2014) evaluated the use of the iPad in the acquisition of requesting skills for children with Autism Spectrum Disorder. Their results showed that training with device was effective for this purpose. Still another study (Waddington et al., 2014), found that three young children with autism spectrum disorder learned to perform a three-step communication sequence using an iPad.

The last decade has seen a dramatic increase in the availability touch devices such as the iPad in homes and schools that are readily accessible to even very young children. There has been considerable discussion in the media as to the value of these technologies for play and learning and as school programs that provide support to preschool children with disabilities and their families are considering the iPad as a possible tool for learning, further information on the effectiveness of this tool is required. There is a need to better understand the role of this and other touch-screen technologies in pre-school contexts and their implications for play and learning. This research study seeks to add to the available information through a systematic look at the use of iPads with eight preschool children included in a preschool program and the learning that the children demonstrated.

## Materials and Methods

The central question for the research was: What improvements in early learning skills will preschool children with disabilities in an inclusive school program evidence while using the iPad loaded with early learning apps over a period of 21 weeks? An additional question was explored:

What support is required by the teacher and families to use this tool? The research took place in an inclusive preschool program with eight preschool children ages three to five identified as having significant disabilities. All eight children were all receiving special funding as the result of identified severe learning challenge(s). Each child in the study had the iPad to use at home throughout the study and were required to bring the iPad into the classroom each day. iPads were chosen for this study for several reasons. First, there was quick and easy access to eight iPads for the study. Second, the teacher expressed an interest in learning more about implementing iPads in the classroom and had some experience in the area. Finally, the range and number of early learning apps suitable for this research and available for use on the iPads far exceeded what was available on other mobile tablet devices that were considered.

This study used a mixed-method approach. First, this study can be seen as participatory action research. The research was an interactive inquiry process between the classroom teacher and the researchers to understand the learning that the children demonstrate, how to best implement this technology and the early learning applications in the early intervention environment, and how to effectively monitor the progress the children were making using the applications. There were 6 action cycles over a period of 21 weeks in this research. In Cycle I the teacher, children, and parents were introduced to the tool. This phase was 1 week in duration. In each of the remaining cycles, each cycle focused on a specific area of preschool readiness, and each cycle lasted 4 weeks. The five focus areas were: Cycle 2 play, drawing, tracing, and creating; Cycle 3 fine motor, tracing, and printing; Cycle 4 concepts color and shapes; Cycle 5 counting, number recognition, and number concept; and Cycle 6 alphabet recognition, letter sounds, printing letters, and early literacy.

The research followed the following procedure. At the beginning of each cycle, the research members reviewed each child’s progress on the iPad and how the iPads were implemented in the classroom. They then planned the course of action for the next 4-week cycle and identified which early learning applications would be used, specific to the focus of the cycle. 6–10 apps were loaded onto each iPad. These apps ranged from simple activities that focused on the skills for the cycle that would be easy for the children to engage with, to more advanced apps that would extend their skills in that specific area. Apps related to the previous cycle were also removed at that time. Before each child received the newly loaded iPad, an informal criterion-based assessment based on the skills related to the focus of the cycle was completed. This assessment focused on the skills in that specific cycle. For example, prior to beginning the cycle that focused on the concepts of shapes and colors, each child’s receptive and expressive knowledge of all of the shapes and colors that would be covered was assessed in a one–one session. Each child was then introduced to the apps for the cycle where the children were encouraged to open and try each app for a few minutes. The children then had the iPad to use at home and at school. Student use of the iPads and apps was observed three times a week a school throughout each cycle. The students used the iPads in a learning center at preschool during play time. At the end of the cycle, each child’s learning related to the cycle was reassessed using the same informal criterion-based assessment and the team used information gathered in the cycle to assist in the planning of the next cycle.

Second, this study utilized a multiple case study design. Multiple-case design, or collective case design, refers to case study research in which a number of instrumental bounded cases are selected to develop a more in-depth understanding of the phenomena than a single case can provide (Chmiliar, 2010). The unit of analysis in this multiple-case approach was each student participating in the study. As the purpose of each case study was to gather comprehensive, systematic, and in-depth information about each case, each case included: pre and post semi structured interview data with parents and teacher; observations of each child using the iPad in the classroom three times a week; and informal criterion-based classroom assessments before and after each cycle. The data was organized into a comprehensive description that includes all of the major information that was then edited, parts fitted together, and organized topically. Each individual case study consists of a description of the child’s experiences with the iPad focusing on the child’s improvements in learning that were demonstrated throughout the research and the challenges that were faced and overcome. Finally, the case studies were integrated across cases, exploring the common threads and differences between the children. A description of parent and teacher feedback is also provided. Patterns in the data were compared between the observations by the researcher and assistant of each child in the classroom, the informal criterion-based assessment results, teacher observations in the classroom, and parent observations at home. If an observation or pattern occurred in two or more of the data collection methods it was considered to be reliable.

## Results

### Student Data

The results begin with a case description developed for each child participating in the study. The description of each child focuses on key improvements in learning that were identified and challenges that were overcome derived from the weekly observations, data from the pre and post informal criterion-referenced assessments, and interview data from parent and teacher interviews. These results are summarized in **Table 1** Student Description.

**Table 1:** Description of subjects.

Child	Age	Gender	Learning difficulties	Improvements in learning	Challenges
1	5	Male	Speech and language	Dramatic play	Too attracted to the iPad
			Social interaction	Drawing and coloring	Mother found it difficult to limit use at home
			Behavior	Tracing	
			Play skills	Printing name	
			Attention	Attention to activities Language Attention Independence	
2	41/2	Male	Speech and language Social interaction Behavior Frustration	Engagement in play Tracing Printing name Concepts Letter and word recognition Language Attention Independence	Very frustrated at the beginning Wanted apps to work right away Preferred apps that related to his interests
3	3 1/2	Male	Speech and language Social interaction Confidence Fine Motor	Language Letter recognition Tracing Singing	Needed support to start using the iPad Struggled with using one finger to tap Limited interest in apps that focused on areas that did not interest him
4	4	Male	Speech and language Social interaction Confidence Attention Frustration Fine Motor	Engagement in play Language Puzzle completion Tracing letters Confidence Independence	Reluctant to engage with the device initially Avoided apps he thought were too difficult
5	5	Male	Speech and language Social interaction Behavior Attention	Play Concepts Numeracy concepts Tracing Printing his name Language Book use	Concerns that he might be just memorizing all of the app content
6	5	Female	Speech and language Attention Fine motor Impulsivity	Creativity Concepts Puzzle completion Tracing Printing Letter recognition	Cost of buying child an iPad Parents found it difficult to limit the use of the device at home
7	4	Female	Speech and language Attention Fine motor	Play Language Puzzle completion Letter identification	Difficulty at the beginning paying attention to apps
8	31/2	Male	Speech and language Fine motor skills Attention	Puzzle completion Concepts Counting Language	Struggled with finger control and accuracy initially


Child 1 was a 5-year-old boy with difficulties in: speech and language; social interaction; inappropriate behavior when asked to do something and during transitions; play skills; and attention. This child exhibited many improvements in learning during all cycles of the research. Child 1 enjoyed the play house app and was observed to make up story lines, plan different activities, and started to verbalize conversations. This type of play was not seen during classroom play time. Child 1 created pictures on the iPad with careful selection of colors and content while he continued to just scribble with crayons and paper. He demonstrated substantial improvement in his ability to trace letters and print his name, learned all of the colors and shapes, and learned to count and recognize numbers. Despite the fact that Child 1 has difficulties with attention, he was able to sustain attention on learning activities on the iPad much longer than in the classroom situation. An increase in verbalizations and self-talk were observed as he used the iPad. Unfortunately, Child 1 experienced several health issues and could not complete the research. In an interview with Child 1’s mother, she indicated that the iPad was very useful at home. He used all of the apps independently at home, was very willing to share use of the iPad and show what he was working on, problem solved, and focused for longer periods of time. The mother felt that her child had made huge progress with speech and language with the iPad, and he was now printing letters and his name on the chalkboard at home. In addition, the mother felt that the use of the iPad for toilet training at home was very helpful, as he was very motivated to use the toilet and have the iPad activities as a reward. However, mother reported that Child 1 was very attached to his iPad and it was difficult for her to limit the use of the iPad, particularly since the substantial learning the child was experience was having such a positive impact on behaviors at home.

Child 2 was a 4½-year-old boy with difficulties in: speech and language; social interaction; inappropriate behavior; and a high level of frustration. This child also experienced many successes with the activities on the iPad. Child 2, similar to Child 1 demonstrated an improved ability to trace shapes and letters and learned to print his name in the app and on paper. Child 2 also learned to identify all of the shapes including all of the complex shapes like pentagon, semi-circle, and crescent. Child 2 really enjoyed apps where he could move letters to make works, and put words into sentences. He particularly enjoyed reading books on the iPad, following along with his finger as each word was said out loud, and saying words to himself. Similar to Child 1, Child 2 engaged on a great deal of self-talk during use of the apps on the iPad and an increase in his verbalizations was evident. Similar to Child 1, Child 2 demonstrated increased engagement with the iPad over time. Child 2’s mother was very convinced that the activities on the research iPad were having a positive impact on her child’s learning. She noted that Child 2 was verbally repeating the letters, sounds, words, and even sentences when he played with the iPad, and these words and sentences had even emerged in conversations at the supper table.

Child 3 was a 3½-year old boy with difficulties in: speech and language; fine motor skills; social interaction; and confidence. Although Child 3 also demonstrated many learning gains throughout the research, he struggled with the iPad initially. He was reluctant to use the iPad and required direction on how to use the apps and how to use one finger to navigate and select items. Child 3 did not enjoy the fine motor apps and would only engage in an activity if the focus of the app was car or vehicle related. Toward the end of the research Child 3 started to engage with the iPad more as he found apps that appealed to him. Similar to Child 1 and Child 2, Child 3 learned to identify letters of the alphabet and huge improvement with tracing letters was observed as he went from not being able to trace at all, to tracing with relative accuracy for many letters. Child 3, like Child 1 and 2, was also observed to engage in more and more self-talk as he used the apps. He started to use a greater variety of words and sentence length also increased. In an interview with Child 3’s mother, she indicated that Child 3 did not use the research iPad at home that much as they had an iPad at home that the child preferred to use with his games and train videos on it. When Child 3 started singing at home for the first time at home, his mother indicated that he was singing songs he was playing with on the iPad. Like Child 1 and Child 2, Child 3 demonstrated increased attention with some activities on the iPad particularly when reading books on the iPad versus print.

Child 4 was a 4-year-old boy with difficulties in: speech and language; fine motor skills; confidence; social behavior; attention; and frustration. Similar to Child, Child 4 was initially reluctant to engage with the iPad. He did not like to engage with apps that he perceived to be a little difficult for him. Once the app was introduced to him and he had a chance to try it a couple of times with help he was more likely to independently choose to use the app. Initially, Child 4 was only independently using one or two apps. About half way through the research it was noted that Child 4 opened and used all of the apps. Like the previous three participants, Child 4 improved his tracing skills substantially and went from not being concerned about staying on the line to being able to trace all of the letters. Similar to Child 1, 2, and 3, Child 4 was observed to participate in increased self-talk and verbalizations as he played in the apps. In an interview with Child 4’s mother, she indicated that her son was not “into the iPad at first and preferred to play outside.” Her son was quite frustrated with apps that did not work immediately for him but was more confident after he played with them with his brother. She reported that at some point his use of the iPad and “his language exploded.” She was convinced that he was imitating the voices in the apps. In her opinion, her child preferred apps that were related to things that he likes such as trains, trucks, and superheroes. She was quite happy that he was now using the iPad independently.

Child 5 was a 5-year-old boy with difficulties in: speech and language; social behavior; behaviors such as following directions and transitions; and attention. Similar to Child 1 and 2, Child 5 engaged in independent and appropriate digital play on the iPad although he typically did not engage in social or constructive play in class. Child 5 also made many learning gains throughout the research. Improvements in tracing and puzzle completion were observed, as well as in shape recognition, counting and number concept. Similar to the previous participants, Child 5 made significant gains in tracing letters and in letter recognition. He learned all of the letters, the sounds of the letters, and had memorized many of the sentences in the apps. And similar to the previous participants, an increase in verbalizations was observed. In an interview with both the mother and the father of Child 5, they indicated that their child had made “incredible” learning gains using the iPad. They had struggled at home to get their child to participate in any learning activities including reading stories to him. Now their son loves the book apps that tell a story and will tell the story back to them. They noted that he had learned to trace all of the letters, could count to 100, learned to write his name, and learned all the shapes. They also indicated that they felt his vocabulary had really increased.

Child 6 was a 5-year-old girl with difficulties in: speech and language; attention; fine motor; and impulsivity. Child 6 was very familiar with the iPad at the start of the study and she used the iPad in very different ways than the other children in the study. Child 6 changed the picture on her screen and every week a new creation was on display. This child created many stories, pictures, and videos independently. In addition to her creations, Child 6 was observed to make many learning gains. She demonstrated improvements in puzzle completion, shape and color recognition, and counting. Similar to the previous participants, Child 6’s ability to trace letters improved. Her ability to stay on the line while tracing letters did not change, but she learned to trace the letters in correct and organized way. In an interview with Child 6’s mother and father, they indicated that their daughter enjoyed using the iPad to take pictures, record her voice, and make video movies. The parents felt that she had explored all of the apps and indicated that they had observed improvements in printing, recognizing letters, counting, and puzzle completion. Unfortunately, they reported that they had difficulties getting the iPad away from her and struggled to set parameters around the iPad use. They also indicated that although they would like to purchase an iPad to continue their daughter’s learning, they had concerns about the cost of the tool.

Child 7 was a 4-year-old girl with difficulties in: speech and language; attention; and fine motor skills. Similar to Child 3, 4, and 5, Child 7 needed help to get started with the iPad. Although she was interested in the iPad, all she was able to do was tap the screen over and over without even looking at what she was doing. Over time her ability to attend to learning tasks on the iPad improved substantially. Similar to the previous participants, Child 7 demonstrated a number of learning gains in many areas. Child 7 went from not being able to complete any puzzles to independently completing 32 piece interlocking puzzles. Similar to the previous participants, Child 7 made considerable gains in tracing letters and learned to print her name. She also made learning gains in recognizing colors and shapes, counting, and number recognition, letter recognition and sounds. Similar to Child 1, and 2, Child 7 demonstrated an improved ability to focus and maintain attention when working on activities. Unfortunately a parent was not available for an interview at the conclusion of the research.

The final case, Child 8 was a 3½-year-old boy with difficulties in: speech and language; fine motor skills; and attention. Similar to Child 3, 4, and 7, Child 8 was initially a little reluctant to use the iPad at the beginning as he struggled with the fine motor apps due to very poor finger control. Child 8 demonstrated learning gains in a number of areas. He learned to independently complete 12 piece interlocking puzzles, learned to recognize a number of shapes and colors, and made significant gains in counting. Similar to the previous participants, Child 8 demonstrated an increase in verbalizations and ability to maintain attention to learning tasks. During the final interview with Child 8’s mother, she indicated that they did not use the iPad that much at home, but used it a lot as they traveled in the vehicle and at hockey practice. She felt that her son really liked the action and noise in the apps and particularly enjoyed the interactive books. Similar to many of the other parents, the mother felt that the iPad use had a huge impact on her son’s language. He was using a much wider range of words at home and she had noticed that he was using the same inflection in his voice as on the apps.

In summary, all of the participants in this research learned how to use the iPad independently. The majority of the eight children were able to learn how to use the iPad immediately. The other children demonstrated some reluctance initially to use the iPad because they had difficulties with fine motor skills and using their finger to touch and navigate, because they were not able to maintain attention on the screen, or because they were not interested in the content of the apps. Each of the students that demonstrated difficulties were able to overcome their difficulties in a short period of time with verbal directions, modeling, positive feedback, and practice. As the research progressed it was evident that all of the students were enjoying their learning activities on the iPad and three of the eight students were observed to be able to sustain attention in the activities for longer periods of time. All of the students demonstrated learning gains in a number of areas and all of the children demonstrated improvements in their ability to trace letters and print their name. Several of the children learned the letters of the alphabet, the sounds the letters make, some simple words, and two of the students were very interested in reading sentences on the apps. All of the children demonstrated increases in self-talk while they played, and increases in vocalizations and vocabulary were observed at school and by the parents at home. Two of the children demonstrated a range of play skills on the iPad such as creative play or construction that they were not able to demonstrate in play time in the class.

### Parent Feedback

All of the parents interviewed in the study spoke positively about the iPad as a learning tool. One parent commented, “We love having the iPad… hugely beneficial.” Another parent said, “This is a good tool… it is a great tool to reinforce things, it is easier to manage their learning, easier when the child thinks it is a game and they can do it themselves.” Yet another parent agreed with this, “He does not think he is learning, he thinks he is playing.” In one interview, a mother and father commented that the iPad, “May be better than therapy – it is play, there is no judgment, he feels included and he is using the same device as mom and dad.

During this research project, the parents were provided with different kinds of support to help them use the iPad at home with their child. At the beginning of each cycle, the parents were provided with a list of the apps that were loaded onto the iPad for their child. A brief description of each app was given. Most of the parents indicated that the newsletter was more than enough information for them and they were able to look at and understand the apps based on this information. Several parents indicated that the newsletter was not quite enough information for them to understand how all of the apps worked. In addition to the newsletters, an afternoon workshop was held for the parents to show how many of the apps worked. There was also a demonstration of how to use the apps for developing books and videos. One parent indicated that the workshop was very helpful for her and without the workshop she would most likely not have tried to create a digital story with her son although she was still working on how to do this. One parent said that the workshop was good exposure to all of the apps; otherwise she would not have looked at and tried all of the early learning apps. Many of the parents indicated that they were loading the apps used in the research onto their own devices; one mother reported that she would like to be able to buy an iPad fully loaded with all of the apps from the research study.

There were only a couple of negative comments expressed by the parents during the final interviews. One set of parents were concerned that the iPad was too expensive for them to purchase for their daughter. This was making them feel bad because they had witnessed so many learning gains when their daughter used the iPad and she liked it so much. The other comment is not so much negative as constructive. Another set of parents indicated that they would really like to see the curriculum coordinated with the apps. Although the themes of the apps and the themes for learning in the classroom were similar, they would have liked specific information as to how the apps related to classroom learning objectives and their child’s individualized learning plan.

### Teacher Feedback

In the final interview with the classroom teacher, she reported that she was happy with the learning that the children displayed on the iPad. She felt that learning activities on the iPad were for the most part very good and very engaging for many of the children. She was most impressed by the fact that this mode of learning “…seems to work for children that are difficult to reach and teach in other traditional ways.” This may be because the iPad is a “…very powerful tool, multi-modal and attention getting.”

The teacher also indicated a number of concerns with the implementation of iPads in the classroom. Her main concern was with time for planning. She felt that it took a lot of time to set up the iPad with apps and to change the apps for each cycle. A considerable amount of time was involved in finding and selecting which apps to use. There is also additional time required to determine how the apps match up with classroom goals and each individual child’s learning objectives and to set up the iPad with the apps. During the research, difficulties were experienced downloading apps as there was very poor wireless access in the school. All of these issues are a concern for the teacher because time to work on the iPad was taken from planning time for other things in the classroom.

The second significant challenge for iPad implementation in the classroom was the need to monitor the use of the iPad in the classroom. The teacher felt that the use of iPads in the classroom does require supervision as she would want to know how the children were using the iPads. During the research additional staffs were available for supervision. In normal circumstances this support would not be available. A checklist with the children’s names, apps, and skills was set up for the classroom learning center. This type of checklist, if used by the teacher after the research, might help her address this problem.

The teacher reported that there were a number of uses for the iPads in the classroom in her opinion. She could see the iPads best used in a play center with concept related apps similar to how the iPads were used in the research. The iPad could also be used in a therapy model and customized for each child’s needs. At the conclusion of the research, the teacher could also see the importance of involving parents in the use of iPads through parent meetings where they could try different apps and see what apps they might want for their child.

## Discussion

### Child Outcomes

Mobile devices such as the iPad have been becoming more prevalent and these devices are frequently becoming part of the early childhood experience at school and at home. In this study, it was observed that the majority of the children participating learned how to use this device quickly. This finding is consistent with the literature. Couse and Chen (2010) found that preschool children learned to use the iPad quickly and were able to explore independently. Beschorner and Hutchison (2013) observed that young children could use iPads independently with limited teacher involvement. Even young children with disabilities were able to master navigation of the iPad quickly and easily (Chmiliar, 2013, 2014). However, it was observed in this study that several children needed a little more support, direction, and time before they were willing to be fully engaged with the device. Child 3, 4, and 8 lacked the fine motor skills to correctly make choices on the iPad screen and as a result avoided using the iPad because they did not want to make errors. Once they had instruction on how to use their finger to tap and additional practice they were able to use the iPad independently. Child 7 had difficulties attending to content on the screen initially. With prompting she was able to learn how to focus on activities. It cannot be assumed that children already have the skills to use the device, and young children with disabilities who may have fine motor challenges may need additional time to learn how to navigate the device.

The literature indicates that the use of educational technologies in the classroom and at home can result in positive learning outcomes for young children (McCarrick and Li, 2007) and the use of early learning apps on the iPad can help children learn preschool children (Dobler, 2012; Chmiliar, 2013; Beschorner and Hutchison, 2013; Flewitt et al., 2014). Research supports the view that children with special needs – mobile learning can be a part of the solution that can help children with special needs to communicate and learn basic concepts (Kokkalia and Drigas, 2016). In this study, the children displayed learning gains in many areas. The learning in each cycle differed from child to child. For example, all of the children demonstrated improvements in their shape and color recognition, but several children showed substantial learning gains. Child 1 and 2 learned all of the colors and shapes including shapes not yet learned by their peers in the classroom such as crescent, pentagon, and octagon. All of the children improved in their puzzle completion skills. Child 5, 6, and 7 made huge gains in their skills. Child 7 progressed from not being able to complete a simple puzzle to being able to compute a 32 piece interlocking puzzle. All eight children made substantial gains in their letter tracing, and alphabet recognition. Child 7 learned all of the letters of the alphabet and the sounds that each letter makes. Child 5 not only learned all of the letters of the alphabet and the sounds, but also started to read simple sentences. For several children, the greatest learning occurred when there were specific apps available for them to use that appealed to their interests. For example, Child 4 made minimal learning gains in each of the cycles until the cycles on numeracy and literacy. Once he started working on the math apps that included monsters, his interest soared and his skills started to improve at a more rapid rate. Child 4’s mother also indicated that in the literacy cycle her son’s interest in books expanded dramatically and she observed a corresponding explosion of language development related to the books apps. To maximize the learning potential of this device, it may be advantageous to be aware of the child’s interests and match the apps to the child’s interests and developmental level.

One of the reasons that the use of the iPad and learning apps was so successful was that using the iPad can be fun and engaging. All of the students in this study appeared to like using the iPad and found apps that appealed to their interests. Lee (2015) reported that children in a Head Start program found use of the iPad fun and engaging which resulted in high motivation to participate. Increased learning may also be related to the fact that young children are able to use the device independently. This motivation resulted in increased concentration and persistence to tasks (Flewitt et al., 2014). The mobile applications and learning activities on them may increase children’s interest during learning as a result of multimedia elements such contains multimedia elements such as animation, graphic and video encouragement (Kokkalia and Drigas, 2016). In this study, an increase in attention to tasks as the weeks progressed was observed. The children worked for longer on apps, were able to sustain attention to tasks in apps until they were successful. This was even true for children who had significant attention problems in the classroom. One of the areas that several children experienced significant success in on the iPad was with creative and imaginative play. Several children with inappropriate play skills were able to engage in imaginative play in a playhouse app that were not able to engage in this type of play during centre time in the classroom. This was particularly true for Child 1 and 5. Both children engaged in creative play on the iPad that included dressing characters up and dramatizing household activities. This finding is similar to Verenikina and Kervin (2011), who observed imaginative play when preschool children were using the iPad. Digital creative and imaginative play could become a significant part of young children’s lives, and apps may be able to facilitate a learning environment for children who struggle in this area.

A second area where many improvements were observed for some children in this study was in the area of language. In the classroom, a number of children engaged in a lot of self-talk as they played with the apps, labeling items on the screen, imitating the language that they were hearing, or repeating words or phrases being read to them. It was also observed, that with apps where the children could record their own voices as part of the tasks in the app, they spent a great deal of time recording themselves over and over. For example, Child 6 recorded her voice on every item in one app, correctly identifying in a two word sentence the color of the object and the name of the object. Several parents also reported that they had witness a huge change in their child’s language at home and that they were certain the change was the result of playing in the apps as the language being used was very app specific. Child 2 was reported to be repeating letters, sounds, words, and sentences at home. Child 3’s mother reported that he started singing songs from the iPad for the first time. Child 4’s mother reported that his language us at home “exploded.” Child 5 starting telling stories back to his parents that he had read on the iPad. Child 6 narrated movies and showed them to her parents. Finally, Child 8’s mother reported that his language used increased and that she observed that he was using the same voice inflection in the words as in the apps. At this point every few studies had focused on this area. Improvement in language after playing with apps on the iPad was noted by Chmiliar (2013, 2014) and Murdock et al. (2013) noted improvements in play dialogue when children played on the iPad. Flewitt et al. (2014) observed enhanced communication and collaboration when children played with the iPads in centres. This is a very important area to consider. The majority of children in this study had significant developmental delays in speech and language development and had been receiving support for this learning challenge at school from other professionals. Perhaps playing on the iPad with strategically selected apps could positively supplement speech and language programming for children with disabilities.

Although this study produced similar results to other studies in the literature focusing on the use of the iPad by preschool, this study is significantly different from the others in several ways. This research study occurred in the everyday classroom environment and in the home environments of the children. The situation was not contrived and the children interacted with the iPads as play activities with the teacher, researchers, and parents as observers. If iPads are going to be implemented effectively into preschool classrooms, then data and information gathered from studies that bridge the gap between the research environments and the classroom is very important. This study is also different from others in that multiple perspectives were used to evaluate the children’s learning. The researchers carried out systematic observations and informal assessments of the children in the classroom, the parents shared their perspective and observations from the home environment, and the teacher contributed with her observations and assessment data. Contributions from multiple participants resulted in a rich data set and multiple perspectives on each child’s use of the iPad and the learning that occurred

Finally, and perhaps most importantly, the purpose of this research was not to manipulate the environment to test the effectiveness of the iPad in teaching a specific concept or skill. The purpose was to facilitate access to the use of the iPads for young children with disabilities and monitor how they used the iPads and the learning that occurred. Each child could choose the apps that they wanted to engage with and they explored the apps independently in the play center at school and at home. Each child demonstrated substantial learning in one or more cycles in the research. This may be due to the interactive interface and the engaging multimedia apps that capture attention. Or it may be due to the fact that the apps chosen for each cycle were well designed and included continuous schedules of reinforcement. But, the additional element for success may be that the children had choice and control over their activities. They had the power to engage with the apps independently. Child 7 chose to learn all of the letters of the alphabet and their sounds in a 4-week cycle and chose to learn how to complete a 32 piece interlocking puzzle. Child 2 chose to learn all of the shapes and colors in every app, many of which would be considered to be difficult to learn at the preschool level. Several of the children demonstrated significant changes in the language that they were using at home because they were able to choose to imitate and verbalize sounds, words, and even sentences while playing on the iPad. Although this element needs to be explored further before conclusions can be drawn, it may be an important factor for young children with disabilities who often have few ways of having independence and control in their lives.

### Teacher Outcomes

Education for teachers is required in order for this technology to reach its full potential to support learning in the classroom (McManis and Gunnewig, 2012). In this study, the teacher indicated that the amount of time spent to find apps that related to the curriculum and learning objectives for the children was substantial and that it would take away from other types of essential planning. Teachers could benefit from access to information and resources on what apps to use and how they relate to the curriculum. So much time and effort is required to select good apps in each area. Hutchison et al. (2012) supported this perspective, indicating that the learning potential of iPads is directly related to the teachers’ ability to link the use of the iPads to the curriculum. It is important to ensure that apps on the iPads are used to enhance curricular integration and support identified learning goals (Northrop and Killeen, 2013).

The teacher in this study also reported the importance of being able to monitor the use of the iPads by the children in the class. McManis and Gunnewig (2012) recommend that progress monitoring is need to gather information on how children are interacting with the devices in the learning content. They recommended digital portfolios, apps with built in monitoring, and using the recording features in apps as possible tools for this purpose.

### Parent Outcomes

The parents in this study reported that the monthly newsletters really helped them to understand the apps that their child was using on the iPad and many of the parents also indicated that they found the workshop very useful. One mother indicated that she would not have tried the digital storytelling app without the workshop. Several of the parents indicated that they would like to know how the apps on the iPad related to the class curriculum and learning objectives. Northrop and Killeen (2013) also found that use of the iPads provided an excellent opportunity to connect school and home-learning activities.

Several parents in this study found it difficult to manage the amount of time their child spent on the iPad. One parent was very reluctant to limit her child’s access to the iPad as he was learning so many things and his in appropriate behaviors at home were reduced. The parents who set parameters around the use of the iPad at the beginning of the study reported no difficulties. These are results are similar to Verenikina and Kervin (2011) who observed that parents who predetermined and defined the use of the iPad in terms of time had children that were accepting of the limitations set by the parents. In the present study, several parents reported that their child did not use the educational activities on the research iPad very much due to the fact that another iPad or other digital media was available to the child that had preferred games and videos on it. If fun non-cognitive activities are available to the child as an alternative to educational activities—the child will choose the more preferred activities. If learning is a goal for the child, access to non-educational games and videos can be restricted to specific time such as traveling in the car, then fun engaging educational apps will be chosen more frequently.

## Conclusion

Overall, the iPad appears to be a tool that can help to have a positive impact on young children with disabilities in the preschool inclusive classroom. iPad can be used independently by young children with disabilities and this research showed that children with disabilities can learn a range of preschool skills on the iPad. In light of these results, the iPad could be a promising tool to help preschool children with disabilities to learn skills essential in the inclusive classroom.

Although this study is helpful in illuminating the possibilities of the iPad in the inclusive preschool classroom, it has limitations. There were very few participants in this study. The eight children in the study were a very diverse group of children in terms of their ages and how their disabilities manifested in the classroom. In addition, the teacher in this study was very willing and interested in integrating the iPad into the classroom. Other early childhood teachers may not be as willing or as interested in investing the time, energy, or commitment implementation of iPads requires. The parents were also very interested in participating. This may be in part due to the fact that this preschool classroom incorporated parent involvement into their program, but this may not be the same for other classrooms. Due to the very limited scope of the study and the small number of preschool children involved, there is not yet sufficient evidence to determine the best practices of the use of this tool with this population. However, given the positive results that this study produced, the use of this device as an early learning tool for children with disabilities should be explored further.

## Ethics Statement

The study was approved by Athabasca University Research Ethics Board. The teachers and parent signed a consent form after reading an information letter and discussing the research with the researcher. The parents of the children signed the consent form for their child to participate.

## Author Contributions

LC wrote the grant application, received the grant, set up the research, worked with the teachers, children, and parents, analyzed the data, and wrote the paper.

## Conflict of Interest Statement

The authors declare that the research was conducted in the absence of any commercial or financial relationships that could be construed as a potential conflict of interest.
